# Interactions between chytrids cause variable infection strategies on harmful algal bloom forming species

**DOI:** 10.1016/j.hal.2023.102381

**Published:** 2023-01-12

**Authors:** Katelyn M. McKindles, R. Michael L. McKay, George S. Bullerjahn, Thijs Frenken

**Affiliations:** a Department of Ecology and Evolutionary Biology, University of Michigan, 1105 North University Ave, Ann Arbor, MI 48109-1085, USA; b Great Lakes Institute for Environmental Research (GLIER), University of Windsor, 401 Sunset Ave., Windsor, Ontario, Canada N9B 3P4; c Department of Biological Sciences, Bowling Green State University, Bowling Green, OH 43403, USA; d Cluster Nature & Society, HAS University of Applied Sciences, Onderwijsboulevard 221, 5223 DE, ‘s-Hertogenbosch, the Netherlands

**Keywords:** Chytrid, *Planktothrix agardhii*, Competition, Co-infection, Polyculture

## Abstract

Cyanobacteria have a great diversity of natural enemies, such as herbivores and pathogens, including fungal pathogens within the Chytridiomycota (chytrids). While these pathogens have been previously described on a select number of cyanobacterial hosts and are suspected to play a significant ecological role, little is understood about species interactions and how competition between parasites can affect epidemic development and bloom formation. Here, three *Planktothrix agardhii* isolates from Sandusky Bay, Lake Erie (OH, USA) were challenged in monoculture and polyculture against infection by three isolates (C1, C2, C10) of their obligate chytrid fungal pathogen, *Rhizophydiales* sp. The chytrid isolates were inoculated as single isolates or a mixture of up to three different isolates. In monoculture, host isolates were characterized as highly susceptible (*P. agardhii* 1030), moderately susceptible (*P. agardhii* 1808) or mostly resistant (*P. agardhii* 1801). Co-infection of chytrid isolates on the highly susceptible host isolate had an additive effect on chytrid prevalence, leading to a culture crash where 2 or 3 chytrid isolates were present. Co-infection of chytrid isolates on the moderately susceptible and mostly resistant isolates had no effect on chytrid infection outcome or prevalence compared to infection with a single isolate. In polyculture, the effect on host growth was most significant in the single chytrid isolate treatment, which was attenuated with the addition of mixed chytrid treatments. Genetic analysis of the resulting population after the experimental period showed a tendency for the chytrid isolate C1 and *P. agardhii* 1801 to dominate in mixed population samples. Two different interspecific interactions seem to be in play; varied parasite infection strategies allow for the amplification of infection prevalence due to mixed chytrids in a susceptible monoculture, or competition allows for the dominance of a single chytrid isolate in monoculture and the reduction of infection prevalence in a host polyculture. This work thus highlights how interactions between chytrid infections can change the course of epidemic development and harmful algal bloom formation.

## 1. Introduction

The increased prevalence and severity of cyanobacterial harmful algal blooms in freshwater lakes are a result of human driven eutrophication and climate change due to increased nutrient loads and rising temperatures (Paerl and Huisman, 2009; O’Neil et al., 2012). These blooms are distributed globally leading to environmental, economic, and health concerns (Watson et al., 2015). Harmful algal blooms foul the water and reduce transparency, which limits growth and establishment of macrophytes that promote a clear-water phase (Tilzer, 1987). Blooms are known to affect recreational use of lakes, thus impacting economies of coastal communities (Dodds et al., 2009; Hudnell, 2008, 2010;Steffensen, 2008). Further, some bloom forming cyanobacterial species are known to produce a suite of bioactive secondary metabolites including hepato- and neurotoxins which have been linked to disease in humans and death in pets, livestock, and local wildlife (Kurmayer et al., 2016; Huang and Zimba, 2019; Janssen, 2019).

Harmful algal blooms are part of a complex ecosystem composed of one or more phytoplankton species, grazers, bacteria, viruses, and parasitic fungal pathogens. Only relatively recently have phytoplankton fungal pathogens (Chytridiomycota, or chytrids for short) been recognized for their ecological role in aquatic ecosystems through facilitating the trophic transfer of carbon and essential nutrients to zooplankton (Kagami et al., 2014; Agha et al., 2016; Frenken et al., 2017, 2018, 2020a, 2020b; Gerphagnon et al., 2019). Chytrids produce zoospores that can be a nutritious food source to zooplankton as part of the ‘mycoloop’, a concept that links the transfer of nutrients between largely inedible phytoplankton to zoospores via ingestion of chytrid zoospores (Kagami et al., 2007). Further, infections by chytrids on filamentous cyanobacteria cause the breaking of filaments into shorter fragments, also increasing the edibility of cyanobacteria to zooplankton (Frenken et al., 2020b; McKindles et al., 2021a).

The presence of chytrid fungi have been suggested to be linked to seasonal succession of phytoplankton populations (Van Donk, 1989) and population subdivisions of the same species (Sønstebø and Rohrlack, 2011; Weisbrod et al., 2020) as selective parasitism on one host may promote the dominance of non-susceptible isolates. Related, some studies note that increased host diversity can inhibit adaptation of parasites to hosts, thereby decreasing the effect of chytrid endemics on populations (De Bruin et al., 2008; Kyle et al., 2015b; Agha et al., 2018). Most experimental work performed under stable laboratory conditions on cyanobacteria-chytrid model systems has been performed in monocultures of one host exposed to one chytrid at a time. In the field, however, prevalence of infection on cyanobacterial hosts during chytrid epidemics is often far lower as when compared to the lab, probably due to environmental constraints, or due to an interaction between host isolates/chemotypes with different parasite isolates (Kyle et al., 2015a; Rohrlack et al., 2015; McKindles et al., 2021b).

To explore whether the development of chytrid epidemics may indeed be inhibited by the composition of host and parasite isolates, we exposed mono- and polycultures of cyanobacterial hosts to different mixtures of parasite isolates, providing a gradient of diversity among infections. Host and parasite cultures used in this experiment were isolated from Sandusky Bay, a shallow freshwater eutrophic embayment of Lake Erie (OH, USA), which typically supports cyanobacterial blooms throughout May-October dominated by a diverse mixture of *Planktothrix agardhii* genotypes that are susceptible to infections by several genotypes of the fungal parasite *Rhizophydium megarrhizum* (McKindles et al., 2021a, b). We hypothesize that competition for hosts between the different isolates of chytrid parasites infecting them may influence cyanobacterial growth and thus harmful algal bloom formation.

## 2. Materials and methods

### 2.1. Model system description, isolation, and maintenance

Host and parasite cultures used in this experiment were isolated from Sandusky Bay. In brief, *Planktothrix agardhii* 1030 was isolated using material obtained from Sandusky Bay in 2016 (Kurmayer et al., 2004). *Planktothrix agardhii* 1801, 1802 and 1808 were isolated from Sandusky Bay during the summer of 2018 via capillary tube isolation of single filaments (Hoshaw and Rosowski, 1973; McKindles et al., 2021a). Cyanobacterial isolates were grown as unialgal, non-axenic batch cultures in Jaworski’s Medium (JM; Jaworski et al., 1981). The cultures were maintained in 125 mL glass Erlenmeyer flasks at 22 °C. Light was supplied by warm-white fluorescent tubes at a light-dark cycle of 12 h:12 h at a photosynthetic photon flux density (PPFD) of 10 - 20 μmol photons m^−2^ s^−1^.

The chytrid cultures used in this experiment were also isolated from Sandusky Bay, as described in McKindles et al. (2021a). Infected *P. agardhii* filaments from the 2018 sampling season (May-October) were diluted for capillary tube single filament isolation and inoculated on dense cultures of purified *P. agardhii* hosts. Chytrid isolates were transferred and maintained on *P. agardhii* 1802 under the same light and temperature conditions as the *Planktothrix* host cultures for several months prior to the experiment outlined below.

### 2.2. Diversity experimental setup

The experimental design consisted of 16 different treatments in triplicate; 3 different host clones (1030, 1801, and 1808) and a polyculture (1:1:1 mixture) were exposed to 3 different parasite communities with increasing diversity (1 chytrid isolate, a mixture of 2 chytrid isolates, or a mixture of 3 chytrid isolates) and an uninfected control (Table 1). *Planktothrix agardhii* 1030, 1801 and 1808 were chosen as representative isolates for different stages of Sandusky Bay harmful algal blooms. We focused on their similarity in growth rate, potential as a host for the chytrid isolates, differences in microcystin production and year isolated: *P. agardhii* 1030 produces microcystins (MCs) and was isolated in 2016, P. *agardhii* 1801 does not produce MCs and was isolated in 2018, and *P. agardhii* 1808 produces MCs and was isolated in 2018 (McKindles et al., 2021a). Since the chytrid isolates are usually cultured and maintained on a separate isolate, *P. agardhii* 1802, we assumed that the chytrids at the start of the experiment were not adapted to the isolates used and therefore the isolates were all novel but susceptible hosts.

At the start of the experiment, for each treatment, host culture chlorophyll biomass was standardized to 90 relative fluorescence units (RFU) as determined by a Turner Designs TD-700 Fluorometer (San Jose, CA) after which cultures were distributed in triplicate into Nalgene Oak Ridge 35 mL polycarbonate centrifuge tubes (Thermo Fisher Scientific, Waltham, MA) which fit into the fluorometer sample chamber for *in situ* readings. Each experimental treatment tube was mixed manually by inversion and replaced inside the incubator at random each day to minimize the effect of light availability on infection rates and to homogenize cultures. The experiment was performed at 19 °C and 10 μmol photons m^−2^ s^−1^ at a light-dark cycle of 12 h:12 h as previously described (McKindles et al., 2021b) in a temperature and light controlled incubator (Caron Products, Marietta, OH). While this illumination is low for surface exposure in the field, it mirrors the low light penetration found throughout Sandusky Bay and matches with previous experimental conditions (1 m from surface light penetration ranges from 0–19.3 μmol photons m^−2^ s^−1^, average for 2017–2018 field seasons 1.63 ± 3.16 μmol photons m^−2^ s^−1^, unpublished data; McKindles et al., 2021b).

A parasite zoospore suspension was prepared for each of the chytrid isolates by passing infected cyanobacterial cultures three times over a 5 μm nylon mesh filter (MilliporeSigma, Burlington, MA) to remove host filaments. Zoospores were then counted by fixing a small volume of each filtrate to a final concentration of 0.5% (v/v) glutaraldehyde and allowing the zoospores to settle in a 96-well plate (CytoOne, USA Scientific, Ocala, FL) prior to counting with an inverted microscope (VWR, Radnor, PA). Zoospores were standardized to the same density of 300 mL^−1^ in each treatment.

### 2.3. Measurements

Each day a subsample of 200 μL was taken from each replicate and fixed in a 96-well plate (CytoOne, USA) using a final concentration of 0.5% glutaraldehyde. Plates were stored in darkness at 4 °C until analysis and were used to quantify prevalence of infection. Infection prevalence was determined by inspecting ≥ 200 filaments in each sample from which prevalence was calculated by dividing the number of infected filaments over the total number of filaments inspected. Infection was determined by counting *P. agardhii* filaments with 1 or more sporangia attached to either terminus since infections only occur on the filament ends and infections are lethal (McKindles et al., 2021a, b). The relative change in biomass per experiment unit was determined in each replicate *in situ* every other day on the Turner Designs TD-700 Fluorometer which measures relative chlA fluorescence using internal standards and the utilization of a solid standard for in-vivo freshwater and high blue green algae samples (Turner Designs, 7000–994, San Jose, CA).

### 2.4. Genetic quantification of host and parasite composition

At the end of the experiment (after 21 days), 20 mL of each replicate were concentrated onto 0.22 μm Sterivex cartridge filters (MilliporeSigma, Burlington, MA). Sterivex filters were stored at −80 °C until extraction with the DNeasy PowerWater Sterivex DNA Isolation Kit (Qiagen, Germantown, MD) following the manufacturer’s instructions. DNA quantity and purity were checked using a Quantus Fluorometer (Promega, Madison, WI) and the associated QuantiFluor ONE dsDNA System kit (Promega), per manufacturer’s instructions.

Molecular quantification of total host and total chytrid was performed using previously described primer sets and g-block standards (*Planktothrix agardhii* rpoC1_Plank_F271 and rpoC1_P_agardhii_R472 (Churro et al., 2012) and *Rhizophydium* SB ITS (McKindles et al., 2021b) (Table S1). To generate primer sets that were unique for each host isolate, a whole genome alignment was performed between the three isolates (McKindles et al., 2022) and regions/genes that were found in one isolate sequence but not the others was targeted for primer generation. Genetically, the *Planktothrix* isolates can be placed into 1 of 4 whole genome aligned clusters, with *P. agardhii* 1030 and 1808 in group 4 and *P. agardhii* 1801 in group 1; *P. agardhii* 1030 and 1808 have an alignment percentage of 81.52% to each other while *P. agardhii* 1801 has an alignment percentage of 48.15 and 52.65% to 1030 and 1808, respectively (McKindles et al., 2022). The target region for *P. agardhii* 1801, called AAUma (Table S1) was a two-protein coding region containing a Clan AA aspartic protease and a Uma2 family endonuclease, which were genetically rearranged in the other two isolate genomes, resulting in a unique quantifiable sequence. Two target regions for *P. agardhii* 1030 and *P. agardhii* 1808 were tested for specificity due to several regions of single nucleotide polymorphisms in the genomic sequences: a TPR domain protein and the modification methylase HphIA. Primer sets for both regions tested negative for *P. agardhii* 1801 but failed to distinguish between *P. agardhii* 1030 and *P. agardhii* 1808, which were both positive. Since both isolates were positive, they were lumped together in the genetic analysis, and the TPR domain protein target was chosen due to slightly better efficiency and limit of detection (Table S1). Similarly, the three chytrid isolate ITS sequences (MW192423, MW192424, MW192429) were aligned and regions with multiple single nucleotide polymorphisms were selected for primer design. Only one region was identified as unique enough to generate specific primers, and that was in the C1 ITS region 634:748. Abundances of C2 and C10 were pooled for genetic analysis and assumed to be the remaining portion of the ITS quantification not specifically assigned to C1. Target regions were added to the Integrated DNA Technologies (Coralville, IA) primer designer web application (OligoPerfect Primer Designer; Thermo Fisher Scientific). Selected primer sets were then analyzed by BLAST against the original isolate sequences and general BLAST to assess possible cross-hybridization. Primer sets were tested using DNA extracted from each isolate in a 10-fold dilution series to confirm specificity and efficiency of the primers (Table S1).

Quantitative PCR (qPCR) was performed using 2 μL of each extracted DNA with the PowerUp SYBR Green Master Mix (Thermo Fisher Scientific) and 400 nM of each primer (Table S1). Each sample was run under the same conditions multiple times using the different primer sets. After an initial activation step at 50 ° C for 2 min and a denaturing step at 95 °C for 2 min, 40 cycles were performed as follows: 15 s at 95 °C, 30 s at 55 °C, and 60 s at 72 °C. A melt curve was also performed to ensure a single qPCR product was formed, progressing from 50 °C to 95 °C at an increase of 0.5 °C per cycle. The program was run on a 4-channel Q Real-Time PCR thermocycler (Quantabio, Beverly, MA) along with the Q-qPCR v1.0.1 software analysis program (Quantabio), which was used to determine the cycle threshold (Ct) of each sample. To calculate the ratios of specific isolates of either *P. agardhii* or its chytrids, the delta-delta Ct method was used (Livak and Schmittgen, 2001).

### 2.5. Statistics

Plots were generated and statistics were performed using RStudio version 1.0.153 working on R version 3.6.1 (2019-07-05). Growth rates of *P. agardhii* were calculated from the linear portion of their respective natural log-transformed growth curves vs. time (days). One-way ANOVA followed by TukeyHSD multiple comparison test was performed to examine the statistical significance of variations among means between the chytrid copies mL^−1^ across all treatments.

## 3. Results

### 3.1. In-situ measurements of host growth and chytrid infection

Growth rates of the three uninfected *Planktothrix agardhii* host clones were very similar, with the fastest isolate (1030) growing at 0.128 ± 0.01 day^−1^ and the slowest isolate (1801) growing at 0.110 ± 0.014 day^−1^ (Table S2). The growth rate of the polyculture was also within the same range at 0.109 ± 0.007 day^−1^, indicating the establishment of a stable co-culture.

**Fig. 1.**
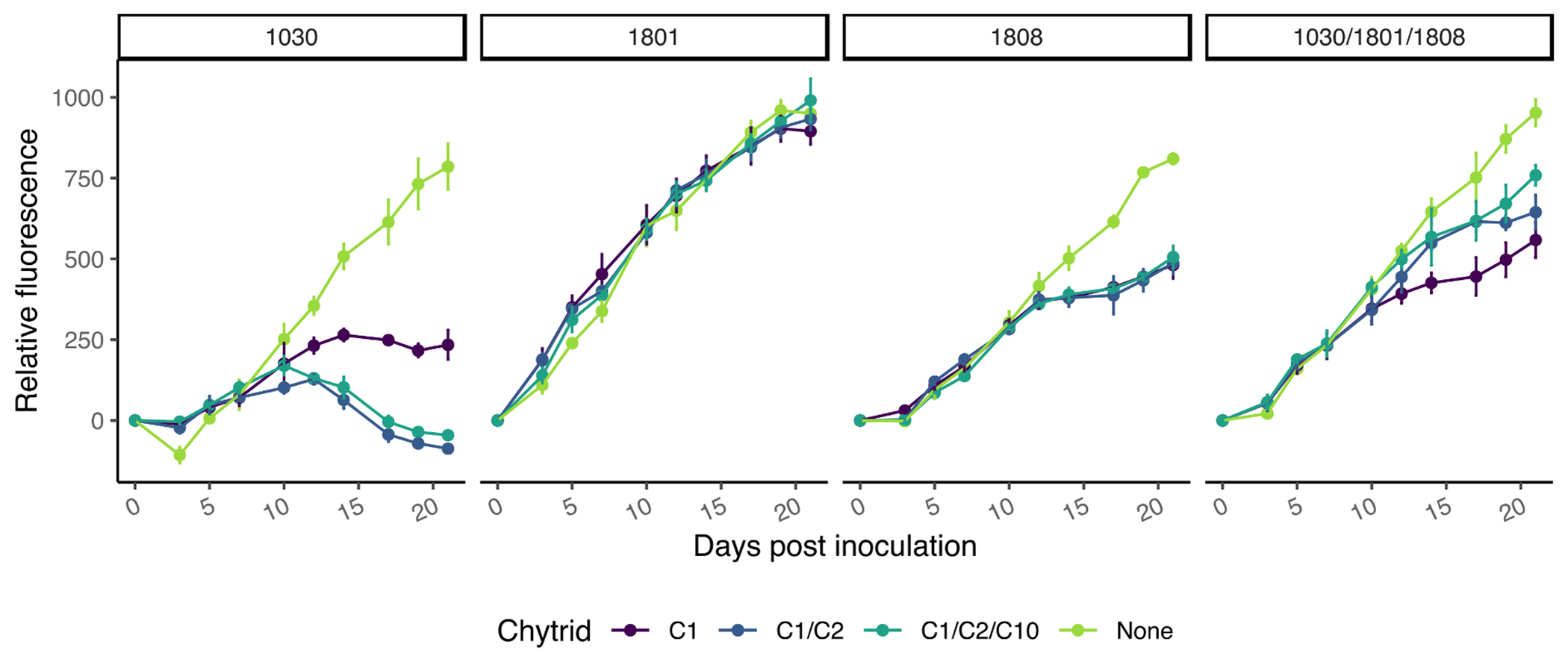
Fluorescence readings in relative fluorescence units (RFU) of *Planktothrix agardhii* growth under uninfected or chytrid infected treatments.

Chytrid infections had very different effects on the growth of the host depending on the host isolate and whether the infection was from a monoculture or mixture of chytrids. *Planktothrix agardhii* 1030 (H1) was the most susceptible to chytrid infection, where the single chytrid infection reduced the fluorescence-measured biomass of the culture by 70.2% compared to the uninfected control and reduced the host growth rate by 45% within 21 days (Fig. 1). Moreover, the fluorescence-measured biomass of both the dual and polyculture chytrid infected *P. agardhii* 1030 were below zero by day 17, indicating a crashed host culture with lower biomass than what was seeded at the start of the experiment. *Planktothrix agardhii* 1801 (H2) appeared to be unaffected by any combination of chytrid infections, as all samples had similar growth rates (0.104 ± 0.007 day^−1^) and maximum fluorescence-measured biomass (947 ± 37.4 RFU). *Planktothrix agardhii* 1808 (H3) was moderately susceptible to chytrid infection, but all combinations of chytrid had the same effect of reducing fluorescence-measured biomass of the culture by 39.4% compared to the uninfected control. Finally, the *Planktothrix* polyculture (H4; 1030/1801/1808) was moderately susceptible to infection with the single chytrid (41.4% reduction in fluorescent-measured biomass) but only slightly susceptible to infections with multiple chytrids (32.3 and 20.3% reduction in fluorescence-measured biomass for two chytrids and three chytrids, respectively).

### 3.2. Infection prevalence

**Fig. 2.**
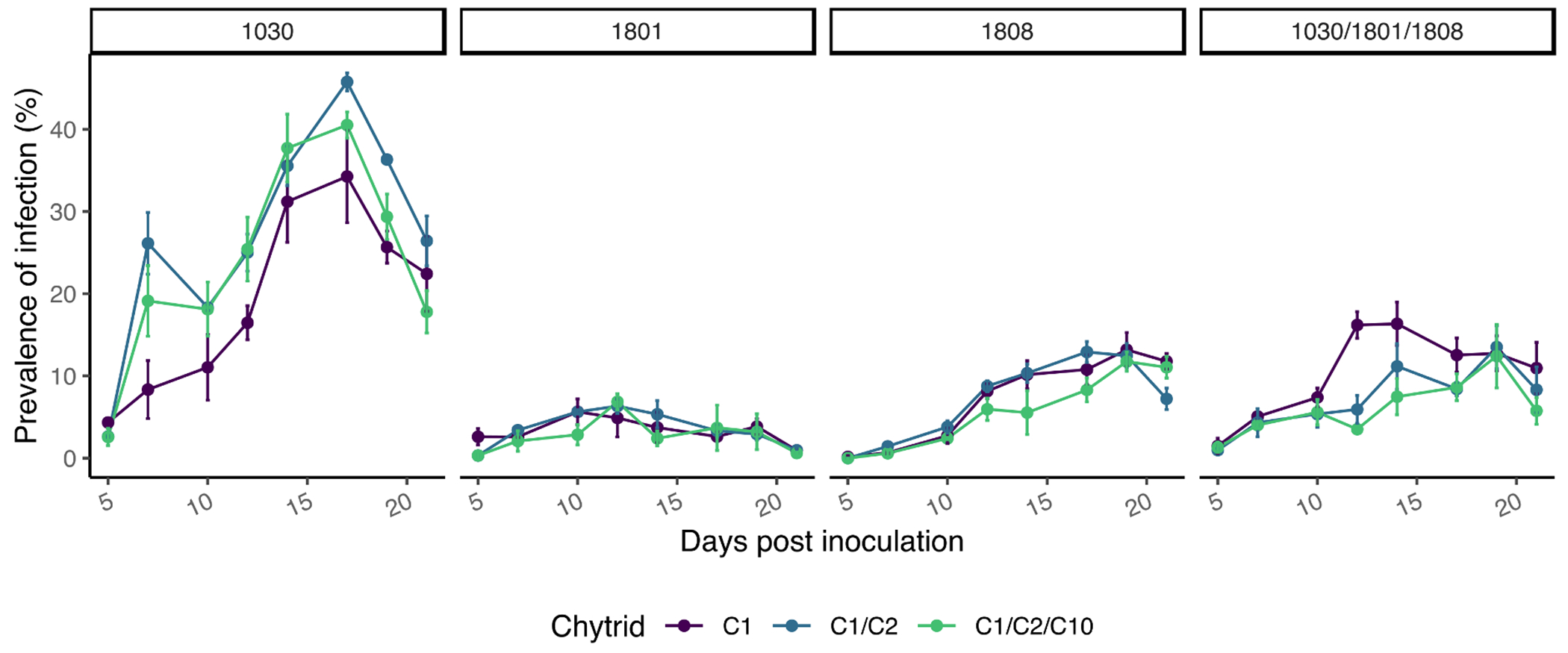
Infection prevalence as measured by the percentage of infected filaments based on the manual counting of 200 filaments. Infected filaments were classified as a filament with one or more attached sporangia at either terminus.

Infections were most prevalent on *P. agardhii* 1030 (H1; Fig. 2), with a maximum infected filament total of 45.8 ± 1.9% when infected with a combination of chytrids C1 and C2 and a maximum infected filament total of 40.5 ± 2.7% when infected with a combination of all three chytrids. When infected with only one chytrid (C1), the maximum infected filament total of *P. agardhii* 1030 was 34.3 ± 9.7%, which is slightly lower than the other infected treatments for this host, but not statistically significant, meaning infection prevalence was similar across all chytrid treatments and no difference was observed in the number of sporangia per infected filament or in the location of infections. Regardless of the infection treatment in *P. agardhii* 1030, chytrid infections reached a peak on day 17. Infections in *P. agardhii* 1801 (H2) were rare, reaching a peak on day 12 at a maximum infected filament total of only 6.3 ± 0.6%. Moderately abundant infections occurred in all infections of *P. agardhii* 1808 (H3) and in the polyculture of chytrid on the *Planktothrix* polyculture (H4). These moderate infections occurred with a peak on day 19 and a maximum infected filament total of 12.7 ± 0.7%. Infection by chytrid C1 on the *Planktothrix* polyculture was both higher (16.2 ± 2.8%) and reached a peak sooner (day 12) than the other infection treatments on the polyculture hosts.

### 3.3. Genetic quantification of host and parasite

**Fig. 3.**
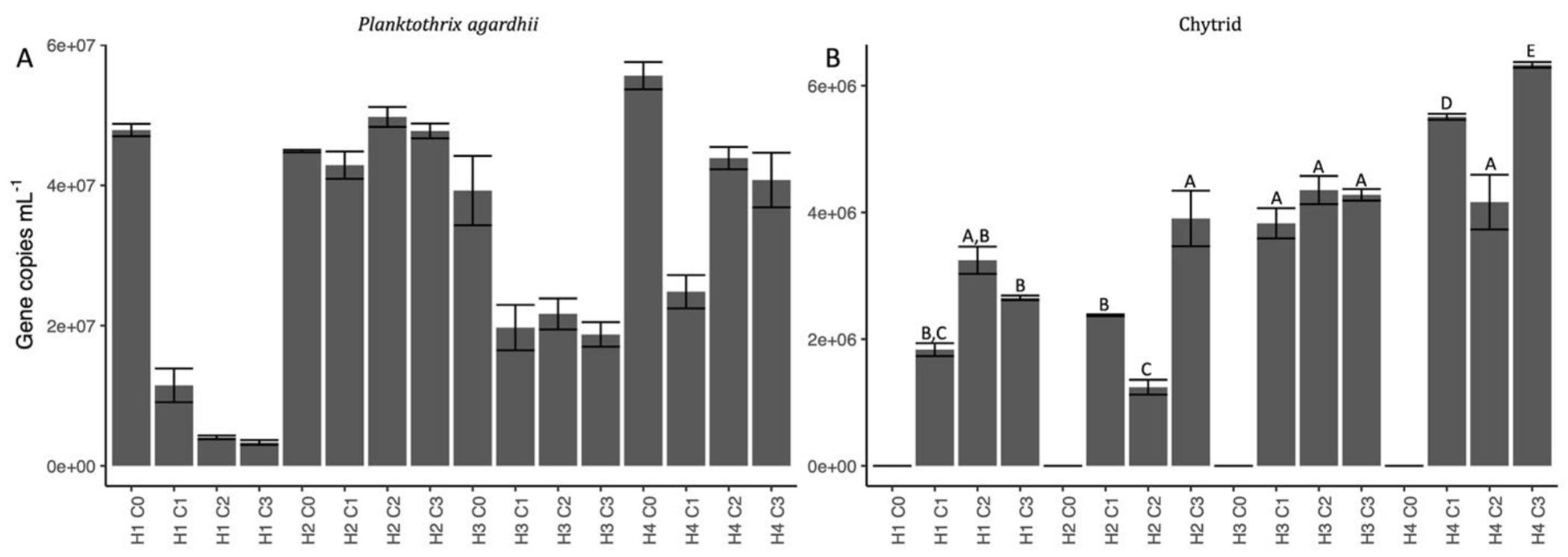
qPCR quantification of (A) total *Planktothrix agardhii* and (B) total chytrid. *Planktothrix agardhii* counts were determined by the single copy housekeeping gene *rpoC*1 and *Planktothrix*-specific chytrid counts were determined by primers for the ITS sequence (Table S1). See Table 1 for treatment nomenclature. (B) One-way ANOVA and TukeyHSD multiple comparison tests group chytrid gene copy mL^−1^. Groups are distinct at *p* ≤ 0.05.

Total quantification of *Planktothrix agardhii* showed similar trends to the fluorescence-measured biomass (Fig. 3a); *P. agardhii* 1030 (H1) *rpoC*1 copy numbers were considerably reduced when infected by one chytrid and further reduced with multiple chytrid treatments, *P. agardhii* 1801 (H2) *rpoC*1 copy numbers remained similarly abundant across treatments, *P. agardhii* 1808 (H3) *rpoC*1 copy numbers were uniformly affected by chytrid infection, and the *Planktothrix* polyculture (H4) was more affected by the single chytrid infection over the multiple chytrid treatments. Indeed, *P. agardhii* 1030 *rpoC1* copy numbers were reduced by 76.0% when infected by C1 and by 91.5 and 93.0% when infected by two and three chytrids respectively. *Planktothrix agardhii* 1808 *rpoC1* copy numbers were reduced by 48.9 ± 3.8% across all three chytrid treatments. Finally, the polyculture *Planktothrix rpoC*1 copy numbers were reduced by 55.4% when infected by C1, but only reduced by 23.9 ± 4.0% under the multiple chytrid treatments. Except for the *Planktothrix* polyculture under multiple chytrid treatments, the genetic quantification of total *P. agardhii* indicated a greater percent biomass loss when compared to the fluorescence-measured values.

Total quantification of the chytrids ranged from 1.24–6.33 × 10^6^ gene copies mL^−1^ and made up 2.5–79.5% of the host population depending on the infection treatment and host (Fig. 3b). Chytrid ITS copy numbers were highest in the polyculture *Planktothrix* host population, averaging 5.33 ± 1.09 × 10^6^ copies mL^−1^ but because the host population was only moderately susceptible to infection, the chytrid population was equated to 15.7 ± 6.4% of the host population. Similarly, chytrid infections in the moderately susceptible *P. agardhii* 1808 were around 4.15 ± 0.28 × 10^6^ copies mL^−1^ and averaged 20.8 ± 1.8% of the host population. Surprisingly, chytrid gene copy numbers were only slightly lower for the least susceptible host isolate, *P. agardhii* 1801, at 2.51 ± 1.34 × 10^6^ copies mL^−1^, but because the host was generally unaffected, these numbers equated to 5.4 ± 2.8% of the host population. Finally, chytrids infective on the most susceptible isolate, *P. agardhii* 1030, made up 16.0% (1.83 × 10^6^ gene copies mL^−1^) of the host population when infected by a single chytrid, which increased to 79.7 ± 0.3% (2.95 ± 0.42 × 10^6^ gene copies mL^−1^) when infected by multiple chytrids.

**Fig. 4.**
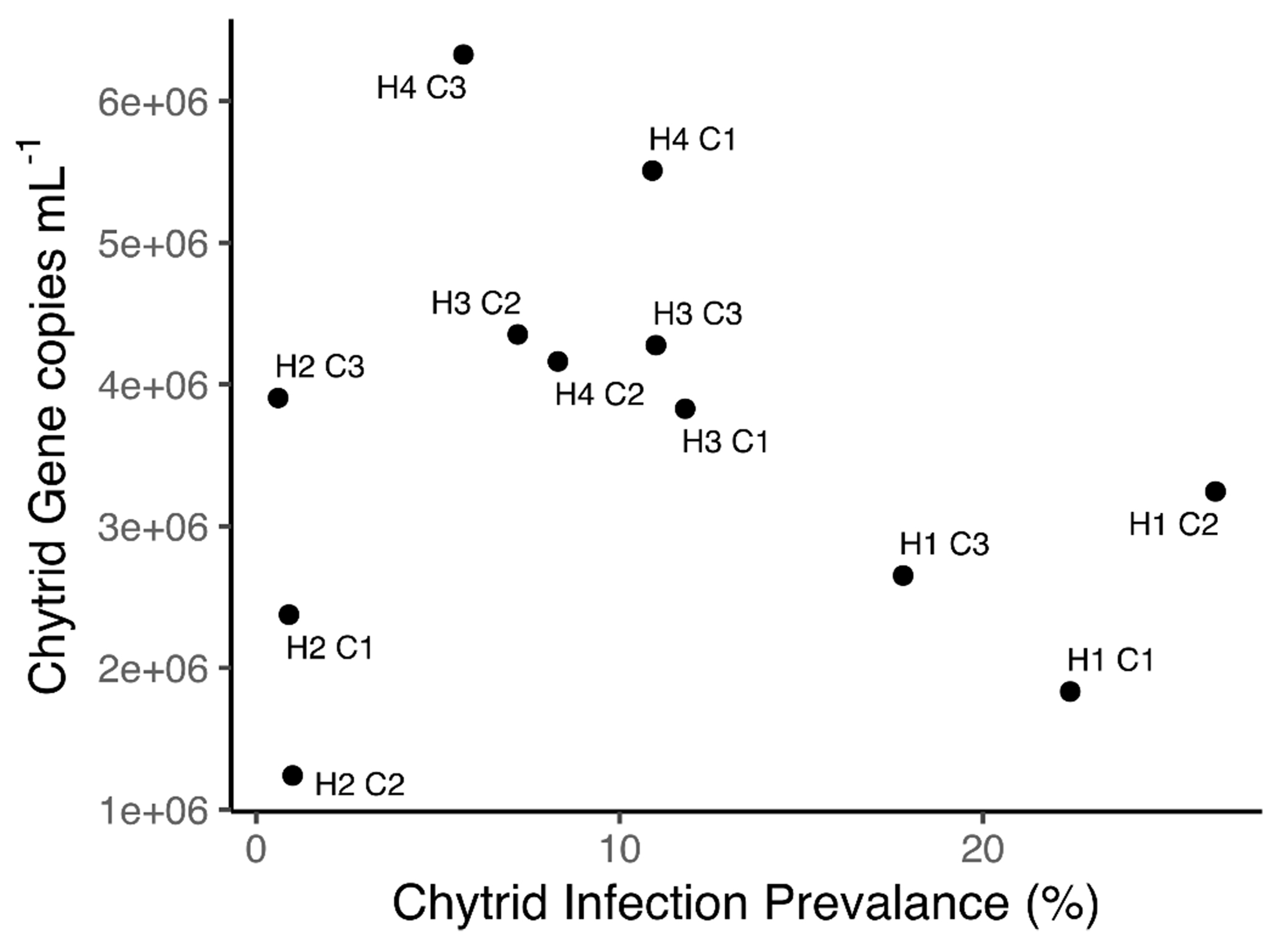
Correlation between gene copy numbers of chytrid ITS and infection prevalence at the end of the experiment. Moderately susceptible hosts (H3, H4) displayed low infection prevalence with higher gene counts, while the opposite was true for the highly susceptible host (H1). See Table 1 for treatment nomenclature.

Patterns of chytrid gene copy numbers per mL did not correlate well with prevalence of infection (Fig. 4). *Planktothrix agardhii* 1030 had the highest prevalence of infection (max 48.5%, 26.4% at harvest), but had lower than average chytrid copy numbers mL^−1^, likely related to a quick die-off of chytrid zoospores after infections peaked on day 17. *Planktothrix agardhii* 1801 infection prevalence was never above 7%, the chytrid gene copies mL^−1^ were quite high depending on the chytrid infection treatment, indicating a larger presence of chytrids (zoospores) than what can be quantified by infected filament counts. *Planktothrix agardhii* 1808 infection patterns matched between infected filament counts and gene copies mL^−1^. Finally, despite infected filament counts that mirrored those of *P. agardhii* 1808, infection prevalence based on chytrid gene copies mL^−1^ were the highest in the *Planktothrix* polyculture.

**Fig. 5.**
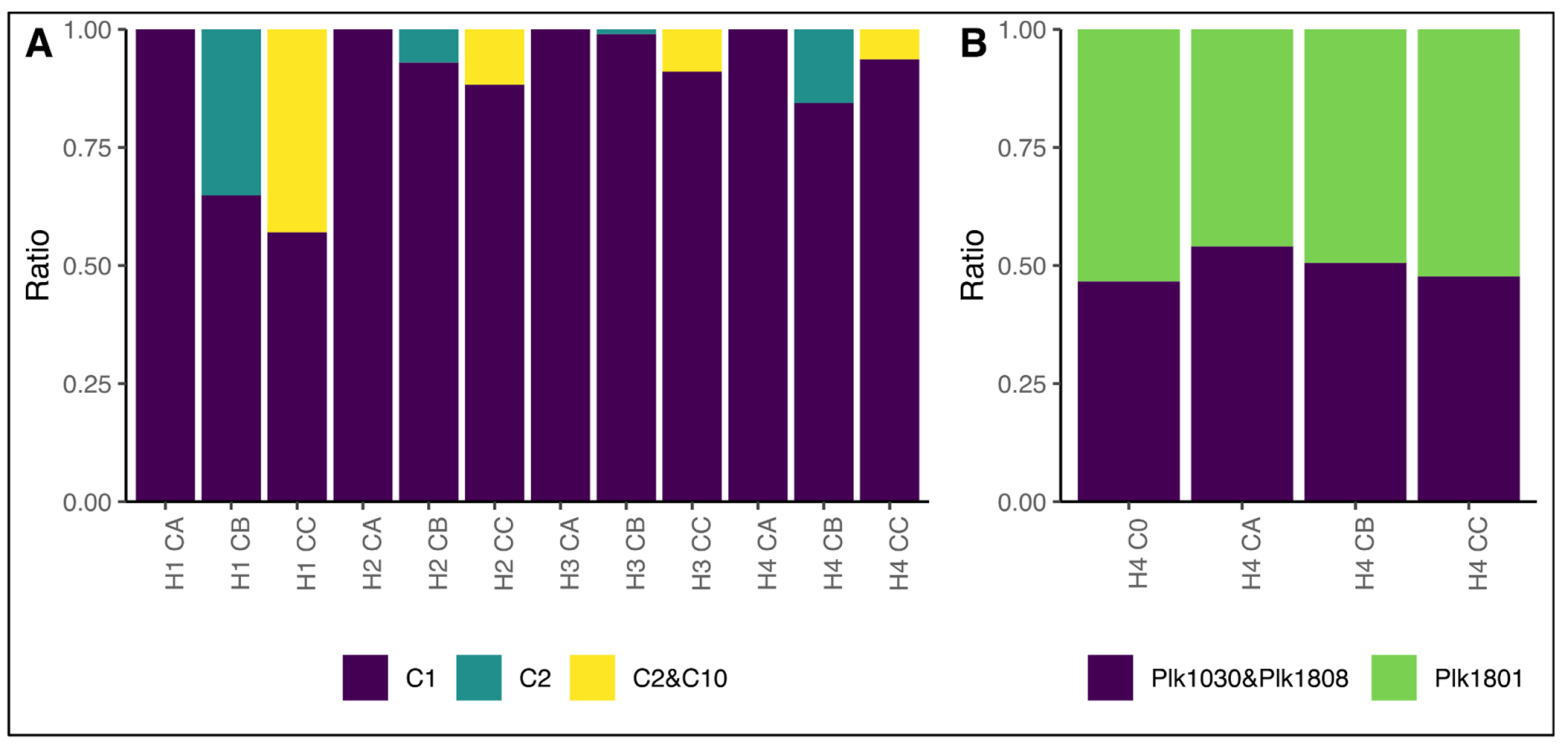
Ratios of host or chytrid isolates in mixed culture as determined by isolate specific primer sets. A. Chytrid ratios in infected samples. B. *Planktothrix agardhii* isolates in mixed culture. See Table 1 for treatment nomenclature.

To better understand the relationship between host or chytrid diversity compared to monoculture treatments, specific primers were used to further characterize dominance. In general, chytrid C1 was the most abundant isolate in the multiple chytrid populations and *P. agardhii* 1801 the most abundant host in the polyculture host populations (Fig. 5a). Except for infections in *P. agardhii* 1030, chytrid C1 out-competed C2 and the combination of C2 and C10, suggesting chytrid C1 to be better adapted to these infection parameters. Alternatively, mixed infections in *P. agardhii* 1030 were not largely dominated by C1 (Fig. 5), which appears to have led to a more aggressive infection as the mixed chytrid infections displayed a greater reduction in fluorescence-measured biomass (Fig. 1) and host gene copies mL^−1^ (Fig. 3a).

## 4. Discussion

Given the complex nature of harmful algal blooms in terms of strain succession and maintenance of multiple isotypes within the same spatial and temporal scale (Welker et al., 2004; Kurmayer and Gumpenberger, 2006; Briand et al., 2008; Wang et al., 2021), we decided to test how diversity in potential hosts and diversity in potential fungal parasites affects the establishment of either species within the bloom ecosystem. Single isolates and a polyculture of three isolates of the cosmopolitan bloom-forming cyanobacterium *Planktothrix agardhii* were tested against one, two, or three different chytrid isolates to determine if increased host diversity and/or competition among the chytrid isolates would change rate of infection leading to a different bloom biomass buildup. The three *P. agardhii* isolates used in this experiment (1030, 1801, 1808) displayed different chytrid infection susceptibility levels, and reacted differently to increased chytrid diversity (Figs. 1, 2). The chytrids, on the other hand, tended to be dominated by one isolate, C1, outcompeting the other two isolates whenever present (Fig 5).

*Planktothrix agardhii* 1030 was highly susceptible to chytrid infection and mixed infections were more effective than the single chytrid (Fig. 1). Indeed, in both multi-chytrid treatments, C1 constituted the smallest majority percentage out of all tested host-chytrid combinations, only making up 64% and 57% of the total chytrid population by the end of the experiment for two chytrids and three chytrids, respectively (Fig. 5). Given the increased prevalence of chytrid infection in *P. agardhii* 1030 with the addition of other chytrid isolates, and the more balanced ratio of chytrid isolates by the end of the experiment, we suspect that this infection dynamic is indicative of variable infection strategies. Unlike the ‘tragedy of the commons’ hypothesis (Hardin, 1968), where direct competition for a host ensures the survivability of a more virulent strain, these data suggest that there might be host partitioning when it comes to chytrid infections under competition. Chytrid C2 infections appear to be more lethal than C1 infections, causing the crash of *P. agardhii* 1030 host cultures (Fig. 1), but C1 appears to outcompete C2 as evidenced by its continual existence and dominance of the chytrid population at the end of the experiment (Fig. 5). Perhaps by not being as lethal, C1 ensures continual survival better than C2. Alternatively, this relationship could be another example of co-infection causing a greater prevalence of infection, as seen in other host-parasite interactions (Mehl and Cotty, 2013; Susi et al., 2015; Diaz-Munoz, 2019). Prior data on the infectivity of individual chytrid isolates on *P. agardhii* 1030 suggests that at a higher initial inoculum of the parasite, C1, C2, and C10 are all lethal resulting in infection prevalence greater than 60% (McKindles et al., 2021a). This points to the idea that any of the three isolates by themselves would have an infection curve like the *P. agardhii* 1030 – C1 curve (Fig. 1), but the addition of another chytrid isolate greatly enhances infection lethality. This effect was not exacerbated with the addition of a third isolate, either indicating that there is a co-infection effect limit, or that the third isolate utilizes a similar infection strategy to C1 or C2 and was thus a competitor.

*Planktothix agardhii* 1801 was largely resistant to chytrid infection, only reaching a maximum infection percent of 6% and decreasing after as the resistant population continued to grow (Figs. 1, 2). Whereas *P. agardhii* 1801 does not produce microcystins, evidence suggests it produces unique secondary metabolites compared to *P. agardhii* 1030 and *P. agardhii* 1808 (McKindles et al., 2022), further complicating the discussion on whether some bioactive secondary metabolites serve a function as an antifungal defense mechanism (Rohrlack et al., 2013; Weisbrod et al., 2020) or not (Agha et al., 2022). An equally interesting trend in chytrid infections on *P. agardhii* 1801 is that the ITS gene copy numbers mL^−1^ for the approximation of chytrid numbers were not statistically lower than the numbers in the other more susceptible hosts (Fig. 3b), perhaps suggesting a shift towards a larger zoospore population, either in size or density, which are able to survive for longer in search for the few susceptible host filaments. Previous work has indicated that under unfavorable conditions, chytrid fungi will produce fewer zoospores while increasing the size of the zoospore to promote longevity (Frenken et al., 2017; Agha et al., 2018).

*Planktothrix agardhii* 1808 was moderately susceptible to infection (Fig. 1) and was affected similarly regardless of chytrid treatment based on both infected filament quantification (Fig. 2) and chytrid ITS gene counts (Fig. 3b). Additionally, specific primers show that C1 excluded C2 and C10 in mixed chytrid treatments (Fig. 5). Together, this suggests only one of the tested infection strategies, the one employed by the C1 isolate, was effective against this host and the addition of other chytrids did not enhance nor inhibit this infection strategy. This relationship could be the result of specific chytrid traits, such as host affinity, generation time, or infective lifetime of the zoospores (Bruning, 1991a, b), or a function of the host, including cell surface traits and the production of chemotactic compounds, as found in other related systems (Moss et al., 2008; Muehlstein et al., 1988). Future work will examine these host-chytrid interaction points to better understand host susceptibility and chytrid infection strategies.

Finally, the *Planktothrix* polyculture is a closer representative of the host-pathogen dynamic most frequently seen in the wild – that is the low prevalence of infection being unable to suppress bloom formation (Kyle et al., 2015b; McKindles et al., 2021b). The polyculture yielded highest abundance of *P. agardhii* 1801 by the end of the experiment (Fig. 5b), which matches the trends seen in monocultures regarding the resistance and susceptibility of each host isolate. If we had the ability to better differentiate *P. agardhii* 1030 and *P. agardhii* 1808 in the polyculture, we would expect to see dominance of *P. agardhii* 1801 with a minor contribution of *P. agardhii* 1808 and a large reduction of *P. agardhii* 1030 as seen in the monoculture host and multi-chytrid treatments. Notably, there was decreased chytrid infection prevalence when more than one chytrid was exposed to the *Planktothrix* polyculture (Fig. 1), suggesting competition among the chytrid isolates in the presence of multiple hosts. This relationship can be partially attributed to the relationship seen by De Bruin et al. (2008) and Agha et al. (2018) where single chytrids can rapidly adapt to single hosts, but do not rapidly adapt to mixed host populations, again suggesting that increased host diversity can hinder parasite adaptation. Since this is the first instance of a multiple chytrid infection on a host polyculture, we can see that in the face of inhibited adaptation, competition further decreases chytrid infection prevalence. In all, the presence of a largely resistant host and a decreased fitness of chytrid isolates when exposed to competition and multiple hosts suggest that large scale, community-wide pathogenesis leading to bloom decline would be rare in cyanobacterial-dominated communities, as described in natural systems (McKindles et al., 2021b; Gsell et al., 2022).

## 5. Conclusions

We explored a variety of host-parasite interactions utilizing a unique system established from ecotypes isolated from a single site as a representative of real-world examples. We found that infections relied heavily on host type, suggesting that there are genetic and or phenotypic differences between the hosts that determine susceptibility. Additionally, differences in chytrid infection prevalence suggest differences in infection strategies, which are successful on some hosts but not others. In summary, we show variable and complex host-parasite interactions require further genetic analysis to understand, which we hope to address in the future.

## Supplementary Material

Mikindles_2023_supplemental

## Figures and Tables

**Table 1:** Experimental design and treatment set-up.

Treatment	Host clone(s)	Parasite isolate(s)
H1 C0	1030	uninfected
H1 CA	1030	C1
H1 CB	1030	C1, C2
H1 CC	1030	C1, C2, C10
H2 C0	1801	uninfected
H2 CA	1801	C1
H2 CB	1801	C1, C2
H2 CC	1801	C1, C2, C10
H3 C0	1808	uninfected
H3 CA	1808	C1
H3 CB	1808	C1, C2
H3 CC	1808	C1, C2, C10
H4 C0	1030, 1801, 1808	uninfected
H4 CA	1030, 1801, 1808	C1
H4 CB	1030, 1801, 1808	C1, C2
H4 CC	1030, 1801, 1808	C1, C2, C10

## Data Availability

Data will be made available on request.
