# Supplementary material for: Interactions between chytrids cause variable infection strategies on harmful algal bloom forming species
Source: Harmful Algae. Author manuscript; Available in PMC 2024 Nov 28. (PMC11603126; doi:10.1016/j.hal.2023.102381)
Supplement: Mikindles_2023_supplemental [file NIHMS2035449-supplement-Mikindles_2023_supplemental.docx]

**Supplementary materials for Interactions between chytrids cause variable infection strategies on harmful algal bloom forming species**

Table S1. Primers and g-block sequences

| Target Organism | Target gene name | Gene (G-) block sequence for generation of standard | Forward primer (5’-3’) | Reverse primer (5’-3’) | Product size (bp) | Efficiency | Standard curve range (copies μL ^-1^) | Ref. |
| --- | --- | --- | --- | --- | --- | --- | --- | --- |
| All *Planktothrix agardhii* | rpoC1 | ATGTGGTGTTAAATCCAGGTAACTATGACGGCCTATCCTATAAACAGTTATTAACCGAAGATACTTGGTTAGAAATTGAAGACCAAATTTATAGTGAAGATTCCACCTTAACCGGAATTGAAGTGGGAATTGGAGCCGAAGCCATTTCCCGTTTGCTCGAAGATATTCCCTTAGAAGAAGAAGCCGAAAGATTACGGGAAGAAATTGCCGTTGCTAAGGGACAAAAACGCGCCAAA | TGTTAAATCCAGGTAACTATGACGGCCTA | GCGTTTTTGTCCCTTAGCAACGG | 224 | 0.971 | 3.866 × 10^9^–38.66 | McKindles et al. 2021b, Churroet al 2012 |
| *P. agardhii* 1801 | AAUma | GATTAATCGTGAATCCAGCAAGTCCTTATTTGGCTAAAAAATCTTTAAAATGAACGAGAGGTATTATGTTAGAAACAGTCACGAGTTTAAATAATTCAATATATCCCACAGAAATTGAGGAAAGTCAGGGAGATATGCC | CGTGAATCCAGCAAGTCCTTAT | TCTCCCTGACTTTCCTCAATTTC | 126 | 0.976 | 5.886 x 10^9^ – 58.86 | This study |
| *P. agardhii* 1030 and *P. agardhii* 1808 | TPR-domain | ACAGTTCGGTTTGCAGGTTGGATCATTGGTCAGCCAATCCCGATGATGGGGGTGCAAATTTTGGGGCATGAGGGACAGGTGATTGAAAATATTCCTGCCCGTTTACCTCGTCCTGATGTAGCTGAAATTT | GTTTGCAGGTTGGATCATTGG | GCTACATCAGGACGAGGTAAAC | 114 | 0.921 | 2.63 x 10^9^ – 26.3 | This study |
| All *Planktothrix*- specific *Rhizophydiales* sp. | Small subunit ribosomal RNA gene, partial sequence : 72 - 311 | TTTTAAGTTGATGCTCCTCGTTGAGCTCACTTGATTCAACTCCCTTTTCACACTTTGTGCACTATGATTGTTTTTTGGGTTGACTGTTACCCATTGGCGACGTCAACCCAGCATTATTTAAAACCATTGTTAATTTGTCTGAATTTTACATATAGTAAATTAAAAACAACTTTTGACAACGGATCTCTTGGTTCTCGCAACGATGAAGAACGCAGCGAAATGCGATACGTAATGTGAATT | GCTCCTCGTTGAGCTCACTT | GTATCGCATTTCGCTGCGTT | 216 | 0.915 | 3.802 ×  10^9^–38.02 | McKindles et al. 2021b |
| Chytrid isolate C1 | Small subunit ribosomal RNA gene, partial sequence : 625 - 791 | CATTTCCTTTTGAACCATGGTCTCCAATCAGATAAGACTACCCGCTGAATTTAAGCATATAACTAAGCGGAGGAAAAGAAACTAACTAGGATTCCCCCAGTAACGGCGAGTGAAGTGGGAATAGCTCAGACTGAAAATCTCCCTTCTGGGGCGAATTGTAGTTTAGA | TTTGAACCATGGTCTCCAATCAG | ACAATTCGCCCCAGAAGG | 151 | 1.05 | 2.379 ×  10^9^–23.79 | This study |

References for Table S1.

Churro, C., Pereira, P., Vasconcelos, V., Valério, E., 2012. Species-specific real-time PCR cell number quantification of the bloom-forming cyanobacterium *Planktothrix agardhii*. Arch. Microbiol. 194(9), 749-57.

McKindles, K.M., Manes, M.A., McKay, R.M., Davis, T.W., Bullerjahn, G.S., 2021b. Environmental factors affecting chytrid (Chytridiomycota) infection rates on *Planktothrix agardhii*. J. Plank. Res. 43(5), 658-72.

Table S2. Calculated growth rates based on natural log (LN) chlA in-vivo fluorescence

| Sample ID | Calculated LN growth rate (day ^-1^) |
| --- | --- |
| H1 C0 | 0.128 ± 0.01 |
| H1 CA | 0.112 ± 0.071 |
| H1 CB | 0.108 ± 0.09 |
| H1 CC | 0.076 ± 0.011 |
| H2 C0 | 0.110 ± 0.014 |
| H2 CA | 0.103 ±0.015 |
| H2 CB | 0.099 ± 0.005 |
| H2 CC | 0.102 ± 0.011 |
| H3 C0 | 0.123 ± 0.013 |
| H3 CA | 0.117 ± 0.03 |
| H3 CB | 0.124 ± 0.075 |
| H3 CC | 0.120 ± 0.037 |
| H4 C0 | 0.109 ± 0.007 |
| H4 CA | 0.089 ± 0.014 |
| H4 CB | 0.105 ± 0.018 |
| H4 CC | 0.104 ± 0.01 |
